# The prognostic value of ultrasound features and parafibromin expression in parathyroid carcinoma

**DOI:** 10.1186/s13023-025-03608-4

**Published:** 2025-03-04

**Authors:** Ruifeng Liu, Liyuan Ma, Yu Xia, Luying Gao, Jiang Ji, Yuang An, Aonan Pan, Nengwen Luo, Yuxin Jiang

**Affiliations:** 1 Department of Ultrasound, State Key Laboratory of Complex Severe and Rare Diseases, Peking Union Medical College Hospital, Chinese Academy of Medical Sciences and Peking Union Medical College, Beijing, 100730 China; 2 Department of Ultrasound, Peking University Shenzhen Hospital, Shenzhen, China; 3 Department of Ultrasound, Peking Union Medical College Hospital, Chinese Academy of Medical Sciences & Peking Union Medical College, 1 Shuaifuyuan, Dongcheng District, Beijing, 100730 China

**Keywords:** Parathyroid carcinoma, Ultrasound, Parafibromin, Prognosis

## Abstract

**Objectives:**

To investigate prognostic factors related with parathyroid carcinoma (PC) based upon ultrasound (US) parameters and parafibromin expression.

**Methods:**

Between 2000/01 and 2022/07, thirty-four PC patients with detailed preoperative ultrasonography were enrolled in the research. Immunohistochemical staining of parafibromin was performed on pathological samples of these patients. Based on the expression of parafibromin, the cases were divided into a positive control group (parafibromin expression ≥ 10%) and a negative experimental group (parafibromin expression < 10%). The ultrasound and clinical features of the two groups were analyzed, and Cox regression was used to identify the independent prognostic factors regarding disease-free survival (DFS) and overall survival (OS).

**Results:**

Among 34 patients with parathyroid carcinoma, 26 (76.5%) were parafibromin-positive, while 8 (23.5%) were parafibromin-negative. The mean follow-up time was 72.6 (11.0-179.3) months. During the overall survival period, 7 cases (20.6%) died, and 9 cases (26.5%) experienced recurrence or metastasis. The median overall survival time (interquartile range) was 65.7 (35.5–89.7) months, and the median disease-free survival time (interquartile range) was 38.2 (22.2–69.7) months. The risk of recurrence and metastasis in the parafibromin-negative group was 5.9 times higher than that in parafibromin-positive group (95% CI 1.569–22.190). PC patients with calcification on preoperative ultrasonography had a 9.4 times higher risk of death during the overall survival period compared with patients without calcification (95% CI 1.037–85.915). However, parafibromin expression did not show a significant impact on the prognosis of the overall survival.

**Conclusions:**

Preoperative US-detected calcification within the lesion is an independent risk factor indicating the shorter OS for PC patients, while loss of parafibromin expression is significant for indicating the recurrence or metastasis of PC patients.

## Background

Parathyroid cancer (PC) is a rare endocrine disease with recurrence rate ranging from 49 to 82%. Most of the recurrences occurred within 2–5 years after the initial surgery for the primary tumor [1, 2, 3]. Once recurrence initiates, the probability of subsequent multiple recurrences and metastases significantly increases and patients result in death due to uncontrollable complications related to hypercalcemia [4]. Utilizing imaging and pathological information from the primary tumor to predict the prognosis of PC could help to develop a stratified management strategy for postoperative patients.

*CDC73* is a tumor suppressor gene located on chromosome 1q31.2, consisting of 17 exons, and its protein product-parafibromin is composed of 531 amino acids. *CDC73* was initially found to be widely mutated in Primary Hyperparathyroidism-Jaw Tumor Syndrome, and according to following studies, a significant correlation was discovered between *CDC73* and tumor metastasis /invasion in the sporadic parathyroid carcinoma [5]. A systematic review on this topic suggests that the absence of parafibromin staining in the nucleus is a risk factor for poor prognosis in PC patients. In patients over 50 years old, the absence of parafibromin is associated with a shorter overall survival (OS) [6]. Imaging features are commonly used to predict the prognosis of patients with certain disease, often yielding favorable results. However, research about ultrasonographic features of parathyroid carcinoma and patients’ prognosis has not been reported. Considering the recognized value of parafibromin staining in the diagnosis and prognosis of PC, it is worth exploring the combined value of ultrasound (US) features and parafibromin expression for predicting the prognosis of PC patients.

## Materials and methods

### Subjects

Thirty-four patients diagnosed with parathyroid carcinoma at Peking Union Medical College Hospital from January 2000 to July 2022 were included in this study. The histopathological diagnosis of PC conforms to the 2017 WHO criteria. All cases had complete preoperative ultrasound data, biochemical test data, and immunohistochemical staining information of parafibromin from postoperative pathological specimens. Disease-free survival (DFS) and OS time were collected from medical records or follow-up phone call. The normal ranges of serum calcium level and serum iPTH level in this study are 2.13–2.70 mmol/L and 12–65 pg/mL, respectively. Recurrence was defined when serum calcium levels exceeded the upper normal limit after the previous remission, excluding other pathological conditions. The study was approved by the Ethics Committee of Peking Union Medical College Hospital (S-K1743), and written informed consent was obtained from the participants.

## Ultrasound examinations

All the ultrasound examinations were performed by radiologists with over 10 years of experience in the field of thyroid/parathyroid disease. The equipment used was either a 7-15 MHz linear array probe from the HDI 5000 (Philips Medical Systems, Bothell, WA) or a 5-12 MHz linear array probe from the IU22 (Philips Medical Systems, Bothell, WA). Gray-scale and color Doppler ultrasound images of target lesions were reviewed by two radiologists who had undergone professional training in ultrasound evaluation of parathyroid diseases. In cases where there was disagreement between the two radiologists regarding characteristics of a specific lesion, they discussed with a third radiologist with over 20 years of thyroid/parathyroid ultrasonography specialty to determine the final result. The location, size, echogenicity, morphology, boundary, cystic change and calcification of the lesions were recorded according to a predetermined research protocol. The main contents are as follows: location (left, right), size (the maximum diameter measured on the different planes), lesion diameter ratio (DR, the ratio between the lesion’s maximum diameter and minimum diameter on two planes perpendicular to each other), morphology (round or oval, irregular), boundary (clear, infiltrative), and echogenicity of the lesion (homogeneous, heterogeneous).

## Immunohistochemical staining

**Fig. 1:**
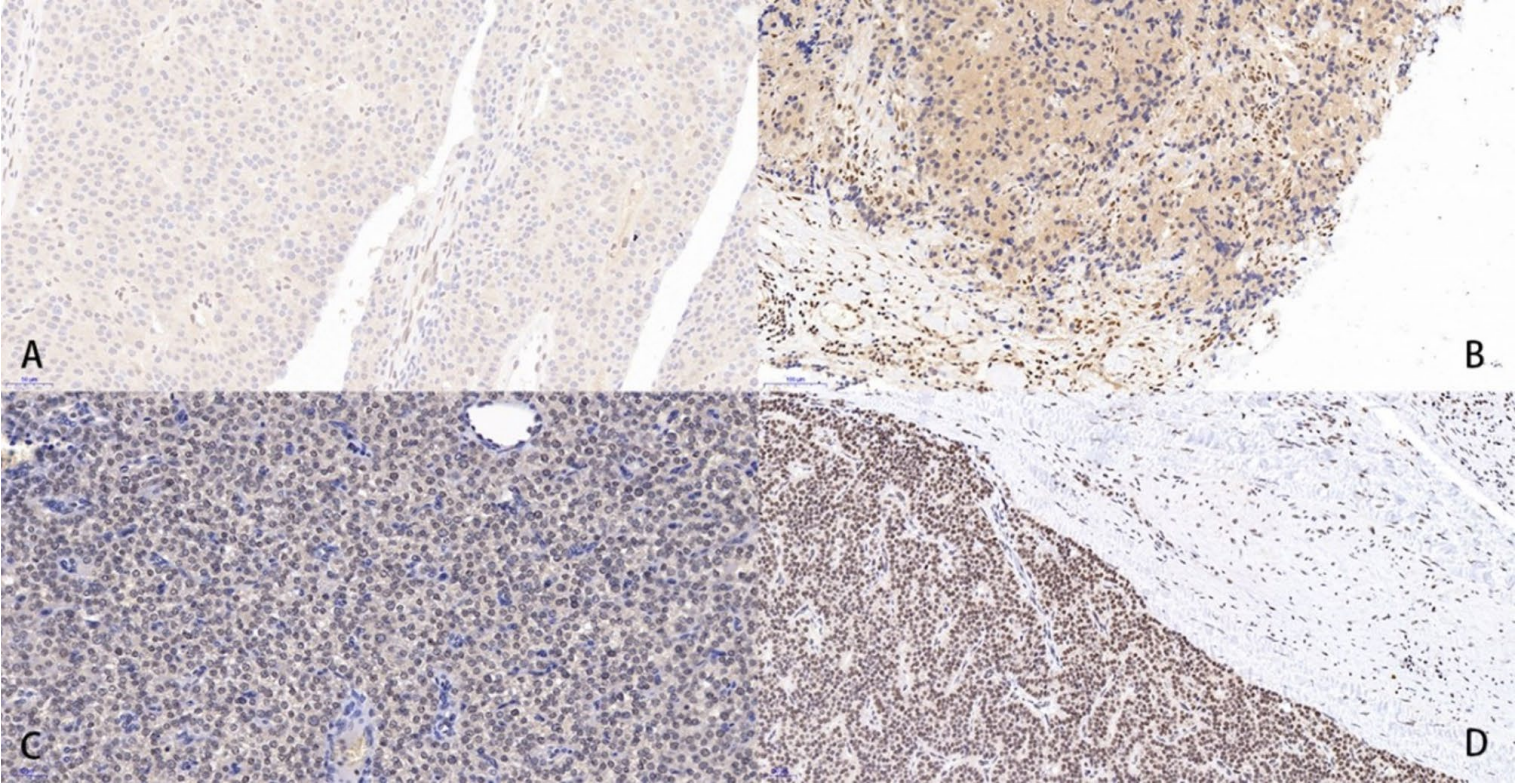
Immunohistochemical staining of parafibromin in parathyroid carcinoma tissue sections. **A**-**B** Parafibromin-negative parathyroid carcinoma tissue, where staining of the tumor cell nuclei is scarcely observed, while the surrounding endothelial cells show brown staining of the nuclei; **C**-**D**: Parafibromin-positive parathyroid carcinoma tissue, where diffuse staining of the tumor cell nuclei is observed

Parafibromin immunohistochemical staining was performed on formalin-fixed, paraffin-embedded tissue samples from primary, recurrent, or metastatic parathyroid carcinoma tumor tissues. The tissue sections were dewaxed, underwent antigen retrieval, and were then soaked in a 3% H2O2 solution to block endogenous peroxidase. Heat-induced antigenic retrieval was performed using an antigen retrieval solution containing ethylenediaminetetraacetic acid (EDTA). After incubating in 3% bovine serum albumin for 30 min to block nonspecific antibody binding sites, the slides were co-incubated with the primary antibodies against parafibromin (ab223840, Abcam, Cambridge, UK) overnight at 4 °C. Then washing the slides with PBS, diaminobenzidine and a secondary antibody working solution coupled with horseradish peroxidase (HRP) (Wuhan Goodbio Technology, China) were used for staining. The sections were counterstained with hematoxylin, differentiated with 0.1% hydrochloric acid ethanol, and rinsed to restore the blue color. They were then dehydrated using graded alcohol solutions and mounted. Each stained section was evaluated by two pathologists without information of the corresponding pathological diagnosis. Parafibromin staining was defined as negative when the staining area of the tumor cell nuclei was less than 10%, while staining was visible in internal controls (vascular endothelial cells and stromal cells). Correspondingly, focal or diffuse staining of tumor cell nuclei (area > 10%) was defined as parafibromin staining positive (Fig. 1).

### Statistical analysis

Data analysis were completed using SPSS 25.0 (Chicago, IL, USA) and R 3.6.3 (R Foundation for Statistical Computing, www.R-project.org). Continuous variables were expressed as mean ± standard deviation or median ± quartile according to the results of the Shapiro-Wilk normality test. Independent sample t-test or Mann-Whitney U test was used for the comparison between groups. Categorical variables were expressed as the number of cases (percentage), and comparison between groups was performed using ANOVA or Fisher’s exact test. Cox regression was used to identify independent risk factors that significantly affect the overall survival and disease-free survival of patients with parathyroid carcinoma. Based on clinical and past research experience, infiltrative margin, calcification, DR value, iPTH, and parafibromin expression status were selected as candidate variables. Kaplan-Meier survival curves were plotted for variables with prognostic value. A two-tailed *p* < 0.05 was considered statistically significant.

## Results

Among all the 34 patients with PC, twenty-six were categorized as parafibromin-positive (male: female, 12:14), while eight were categorized as parafibromin-negative(male: female, 7:1). The median age (interquartile range) of all cases was 49 (37, 59) years, with a mean follow-up time of 72.6 (11.0-179.3) months. There were 7 deaths (20.6%) and 9 cases (26.5%) of recurrence or metastasis during the follow-up period. The median overall survival time (interquartile range) was 65.7 (35.5, 89.7) months, and the median disease-free survival time (interquartile range) was 38.2 (22.2, 69.7) months. There were no significant statistical differences in patient age, gender, iPTH levels, and blood calcium levels between the positive and negative groups (*p* = 0.626, *p* = 0.053, *p* = 0.465 and *p* = 0.542). Similarly, there were no statistical differences in various ultrasound features between the two groups, including lesion size, lesion diameter ratio, morphology, echo heterogeneity, infiltrative borders, calcification, and cystic degeneration (*p* = 0.339, *p* = 0.583, *p* = 0.309, *p* = 0.160, *p* = 0.152, *p* = 1.000 and *p* = 0.693) (Table 1).

**Table 1:** Comparison of demographic, clinical, and ultrasound parameters between parathyroid carcinomas with positive and negative parafibromin staining

	Parafibromin positive (*n* = 26)	Parafibromin negative (*n* = 8)	*P* value
Mean age (years)	49 ± 31	49 ± 16	0.626
Gender (male/female)	12/14	7/1	0.053
Serum iPTH (pg/mL)	1183.5 ± 882	1085.5 ± 1211	0.465
Serum Calcium (mMol/L)	3.3 ± 0.7	3.2 ± 0.8	0.542
Lesion size (cm)	3.9 ± 1.5	3.2 ± 1.2	0.339
DR	1.7 ± 0.7	1.6 ± 0.8	0.583
Shape (irregular/oval)	21/5	8/0	0.309
Echogenicity (heterogeneous/homogeneous)	19/7	8/0	0.160
Infiltration	18(69.2%)	8(100%)	0.152
Calcification	8(30.8%)	3(37.5%)	1.000
Cystic change	13(50.0%)	5((62.5%)	0.693

iPTH, intact parathyroid hormone; DR, the ratio between the lesion’s maximum diameter and minimum diameter measured in all the ultrasound dimensions

**Fig. 2:**
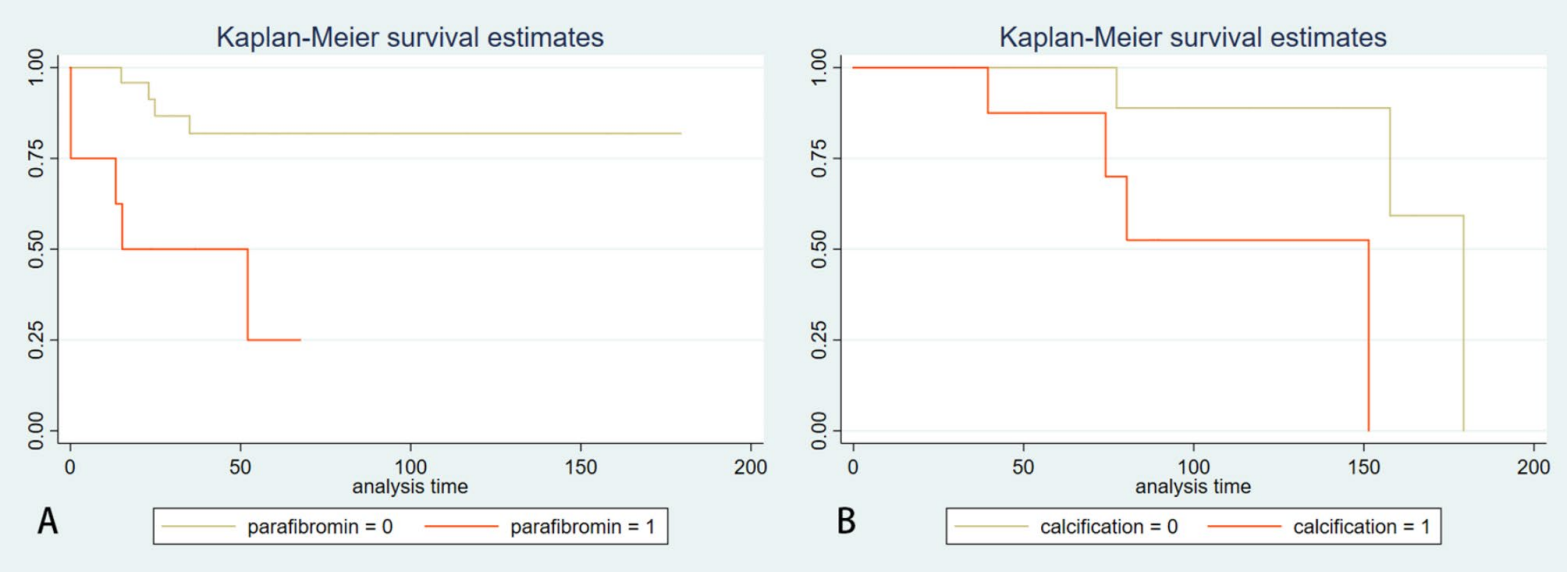
The Kaplan-Meier survival curve analysis. **A** The Kaplan-Meier survival curve shows that PC patients with loss of parafibromin expression (parafibromin = 1) have significantly shorter disease-free survival (DFS) compared with those with positive parafibromin expression (parafibromin = 0) (*p* = 0.009); **B** The Kaplan-Meier survival curve indicates that PC patients with calcification in the lesion (calcification = 1) have significantly shorter overall survival (OS) compared with those without calcification (calcification = 0) (*p* = 0.046)

**Fig. 3:**
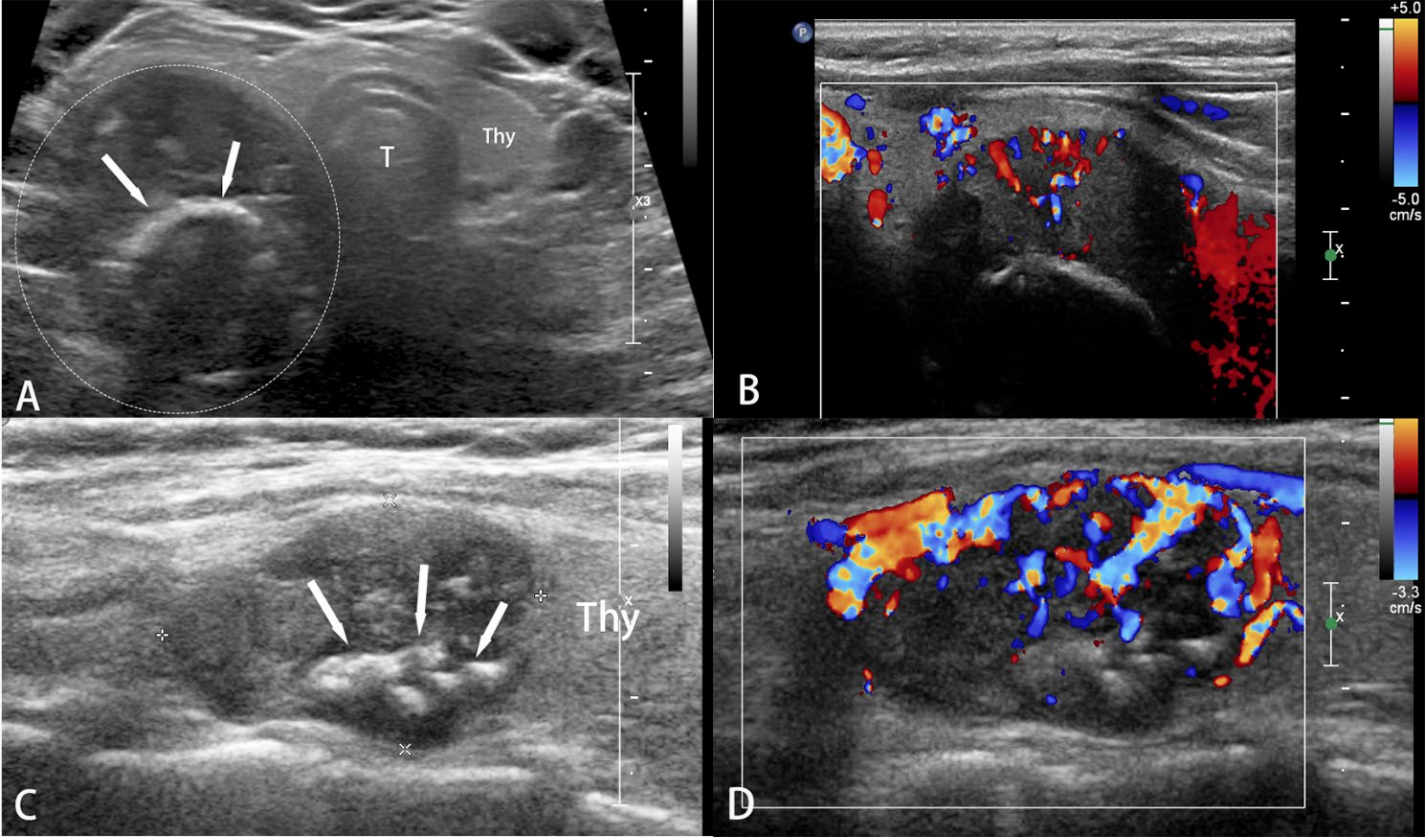
Calcification in the parathyroid carcinoma on ultrasonography. **A** Parathyroid carcinoma of a 45-year-old female with coarse calcification observed in the lesion (arrows); **B** Color Doppler ultrasound shows abundant blood flow in the lesion; **C** Parathyroid carcinoma of a 62-year-old female with multiple calcifications detected in the lesion (arrows); **D** Color Doppler ultrasound shows circumferential and internal blood flow in the lesion. Thy: thyroid; T: trachea

In terms of the disease-free survival, the loss of parafibromin expression is an independent risk factor for recurrence and metastasis in patients with parathyroid carcinoma (*p* = 0.009). The risk of recurrence and metastasis in patients with loss of parafibromin expression is 5.9 times higher than that in patients with evident parafibromin expression (95% CI 1.569–22.190). No statistical differences were calculated in iPTH, DR value, calcification and infiltrative boundaries observed by ultrasound (*p* = 0.096, *p* = 0.552, *p* = 0.362 and *p* = 0.539). In terms of the overall survival, intralesional calcification detected by ultrasound is an independent risk factor for death in PC patients (*p* = 0.046). The risk of death during the overall survival period for patients with calcification in the lesion is 9.4 times higher than that for patients without calcification (95% CI 1.037–85.915). No statistical differences were found in iPTH, DR value, parafibromin expression, and infiltrative boundaries either (*p* = 0.443, *p* = 0.953, *p* = 0.667 and *p* = 0.119) (Figs. 2 and 3).

## Discussion

Due to the rarity of parathyroid carcinoma, single-center retrospective studies are the mainstream of the field. It is difficult for us to fully understand the natural course and prognosis of this disease. The reported 5-year and 10-year survival rates among Caucasian populations are 78–85% and 49–77%, respectively [7, 8, 9, 10, 11]. According to a study by Obara et al. based on the Japanese population, the median survival time after the first recurrence of parathyroid carcinoma is only 7 years, but no data on deaths during the overall survival period is present [12]. Studies based on Chinese population by Song et al. from Shanghai Ruijin Hospital and Hu et al. from Peking Union Medical College Hospital suggest that the 5-year and 10-year overall survival rates for patients with parathyroid carcinoma are 78.9-90.2% and 60.7-77.9% [13, 14], respectively. Thus, precise stratification of prognosis in the early stage is crucial for the postoperative monitoring of parathyroid carcinoma.

Many studies have conducted exploratory analyses on prognostic risk factors for parathyroid carcinoma. The Schulte TNM staging system and Schulte Risk Classification are important contributions, which use the degree of invasion into surrounding tissues and vital organs as the main staging criteria. Both systems indicate a significant increase in recurrence and mortality risk for patients with T3-T4 staging or high-risk classification [15, 16]. Other notable influencing factors include age, extent of surgical resection, preoperative parathyroid hormone and blood calcium levels, parafibromin and Ki-67 expression in pathological specimens. Nevertheless, the potential value of imaging for predicting the prognosis of parathyroid carcinoma is unexplored, but worthwhile. Studies about the correlation between medical imaging features and the prognosis of certain diseases are common and have yielded many meaningful results. Our research also suggests that calcification identified by US within the primary PC lesion is an independent risk factor affecting the overall survival of patients with parathyroid carcinoma. It also reconfirms that parafibromin is an independent risk factor for recurrence and metastasis of parathyroid carcinoma. Both factors play vital roles in the natural course of PC.

Mutations in the tumor suppressor gene *CDC73* represent one of the essential molecular mechanisms about the onset of parathyroid carcinoma. Germline mutations in *CDC73* are closely associated with hyperparathyroidism-jaw tumor syndrome (HPT-JT), which lead to the loss of parafibromin, a kind of protein consisting of 531 amino acids. Parafibromin functions as a tumor suppressor by inducing apoptosis and inhibiting cell cycle-its absence results in overexpression of Cyclin D1 [17]. Ruizhe Zhu et al., through a systematic review and meta-analysis based on individual patient data, found that negative parafibromin immunohistochemical staining is an independent risk factor for recurrence/metastasis (HR 2.73, *p* = 0.002) and death (HR 2.54, *p* = 0.004) in patients with PC, while *CDC73* gene mutations were not significantly correlated with any clinical outcomes [18]. In this study, the loss of parafibromin expression was significantly associated with a shorter disease-free survival but not with the overall survival. Davies et al. suggested that parafibromin can be present in either the nucleus or cytoplasm of chief cells of parathyroid tumor [19]. Inspired by facts stated above, we raise the hypothesis that the localization of parafibromin may influence the prognosis of PC patients in different ways, which represents a direction for future research.

Tumor calcification is a pathological mineralization process that shares many similarities with physiological mineralization (such as bone formation and remodeling). Existing research regarding this topic is not consolidated, and there are many controversies concerning the chemical composition of deposited minerals, crystal characteristics, participating cells and intracellular regulation, gene expression characteristics, mineralization timeline, and other aspects. Nevertheless, some studies suggesting that deposited minerals affect tissue interactions, thereby participating in disease progression, provide valuable insights for us [20]. Calcification within parathyroid lesions is relatively rare, and there is no study reporting its formation mechanism or prognostic impact to our knowledge. For discussion, we can refer to the field of breast cancer microcalcifications, which has yielded significant findings. It assists in exploring the possible reasons of the poor prognosis of PC patients with calcification in the lesion [21]. The chemical substances that constitute breast cancer microcalcifications mainly include calcium oxalate and calcium phosphate, with disparities in molecular formulas, such as (Ca)10(PO4)6(OH)2, Ca3(PO4)2, Ca8H2(PO4)6·5H2O, etc [22, 23]. The microscopic differences behind the similar imaging manifestations may account for the heterogeneity in the prognosis of patients with the same characteristic-calcification. The formation of calcification can be understood as an imbalance between promoting and inhibiting factors caused by changes in the microenvironment. Factors promoting calcification include overexpression of the *BMP2* gene, inflammation, oxidative stress, necrosis, and metalloproteinase expression, etc., while inhibiting factors include magnesium ions, osteopontin, etc. The imbalance between them leads to a cellular phenotypic shift towards osteogenesis, resulting in mineral deposition [24]. The relationship between microcalcification and the prognosis of breast cancer is mainly reflected in three aspects: increased risk of death (mainly cast calcification), increased risk of recurrence, and increased *HER2* expression (high consistency among researches) [25, 26, 27, 28]. In vitro and in vivo studies indicate that this may be related to mechanisms such as microcalcification promoting cell mitosis, enhancing cell migration ability, mediating high expression of inflammatory factors, promoting epithelial-mesenchymal transition, and regulating immune monitoring [29, 30]. Given all above, we speculate that calcification in parathyroid carcinoma may reflect dramatic changes of intratumoral microenvironment-greater oxidative stress, higher inflammation levels, and increased necrosis. Calcification itself may also interact with tumor cells to form a cascade reaction, further increasing tumor invasiveness, leading to a poor prognosis of the patient.

## Conclusions

In conclusion, our study firstly reveals that calcification in the parathyroid carcinoma detected by preoperative US may be related to worse overall survival among PC patients, and we also confirm that the parafibromin significantly affects disease-free survival.

## Data Availability

The datasets used and analyzed during the current study are available from the corresponding author on reasonable request.

## References

[1] Al-Kurd A, Mekel M, Mazeh H. Parathyroid carcinoma. Surg Oncol. 2014;23(2):107–14. 24742584 10.1016/j.suronc.2014.03.005

[2] Shane E. Clinical review 122: parathyroid carcinoma. J Clin Endocrinol Metab. 2001;86(2):485–93. 11157996 10.1210/jcem.86.2.7207

[3] Iihara M, Okamoto T, Suzuki R, Kawamata A, Nishikawa T, Kobayashi M, et al. Functional parathyroid carcinoma: long-term treatment outcome and risk factor analysis. Surgery. 2007;142(6):936–43. discussion 43.e1. 18063079 10.1016/j.surg.2007.09.014

[4] Walker MD, Shane E, Hypercalcemia. Rev Jama. 2022;328(16):1624–36. 10.1001/jama.2022.18331 36282253

[5] Le Collen L, Barraud S, Braconnier A, Coppin L, Zachar D, Boulagnon C, et al. A large extended family with hyperparathyroidism-jaw tumor syndrome due to deletion of the third exon of CDC73: clinical and molecular features. Endocrine. 2021;73(3):693–701. 33999366 10.1007/s12020-021-02756-4

[6] Gao Y, Wang P, Lu J, Pan B, Guo D, Zhang Z, et al. Diagnostic significance of parafibromin expression in parathyroid carcinoma. Hum Pathol. 2022;127:28–38. 35654240 10.1016/j.humpath.2022.05.014

[7] Busaidy NL, Jimenez C, Habra MA, Schultz PN, El-Naggar AK, Clayman GL, et al. Parathyroid carcinoma: a 22-year experience. Head Neck. 2004;26(8):716–26. 15287039 10.1002/hed.20049

[8] Harari A, Waring A, Fernandez-Ranvier G, Hwang J, Suh I, Mitmaker E, et al. Parathyroid carcinoma: a 43-year outcome and survival analysis. J Clin Endocrinol Metab. 2011;96(12):3679–86. 21937626 10.1210/jc.2011-1571

[9] Hundahl SA, Fleming ID, Fremgen AM, Menck HR. Two hundred eighty-six cases of parathyroid carcinoma treated in the U.S. between 1985–1995: a National Cancer Data Base Report. The American College of Surgeons Commission on Cancer and the American Cancer Society. Cancer. 1999;86(3):538–44. 10430265 10.1002/(sici)1097-0142(19990801)86:3<538::aid-cncr25>3.0.co;2-k

[10] Lee PK, Jarosek SL, Virnig BA, Evasovich M, Tuttle TM. Trends in the incidence and treatment of parathyroid cancer in the United States. Cancer. 2007;109(9):1736–41. 17372919 10.1002/cncr.22599

[11] Sandelin K, Auer G, Bondeson L, Grimelius L, Farnebo LO. Prognostic factors in parathyroid cancer: a review of 95 cases. World J Surg. 1992;16(4):724–31. 1413841 10.1007/BF02067369

[12] Obara T, Fujimoto Y. Diagnosis and treatment of patients with parathyroid carcinoma: an update and review. World J Surg. 1991;15(6):738–44. 1767540 10.1007/BF01665308

[13] Hu Y, Bi Y, Cui M, Zhang X, Su Z, Wang M, THE INFLUENCE OF SURGICAL EXTENT AND PARAFIBROMIN STAINING ON THE OUTCOME OF PARATHYROID CARCINOMA, et al. 20-YEAR EXPERIENCE FROM A SINGLE INSTITUTE. Endocr Pract. 2019;25(7):634–41. 30865538 10.4158/EP-2018-0538

[14] Xue S, Chen H, Lv C, Shen X, Ding J, Liu J, et al. Preoperative diagnosis and prognosis in 40 parathyroid carcinoma patients. Clin Endocrinol (Oxf). 2016;85(1):29–36. 26939543 10.1111/cen.13055

[15] Talat N, Schulte KM. Clinical presentation, staging and long-term evolution of parathyroid cancer. Ann Surg Oncol. 2010;17(8):2156–74. 20221704 10.1245/s10434-010-1003-6

[16] Schulte KM, Gill AJ, Barczynski M, Karakas E, Miyauchi A, Knoefel WT, et al. Classification of parathyroid cancer. Ann Surg Oncol. 2012;19(8):2620–8. 22434247 10.1245/s10434-012-2306-6

[17] Li Y, Zhang J, Adikaram PR, Welch J, Guan B, Weinstein LS, et al. Genotype of CDC73 germline mutation determines risk of parathyroid cancer. Endocr Relat Cancer. 2020;27(9):483–94. 32590342 10.1530/ERC-20-0149 PMC8802173

[18] Zhu R, Wang Z, Hu Y. Prognostic role of parafibromin staining and CDC73 mutation in patients with parathyroid carcinoma: a systematic review and meta-analysis based on individual patient data. Clin Endocrinol (Oxf). 2020;92(4):295–302. 31945198 10.1111/cen.14161

[19] Davies MP, John Evans TW, Tahir F, Balasubramanian SP. Parathyroid cancer: a systematic review of diagnostic biomarkers. Surgeon. 2021;19(6):e536–48. 33642204 10.1016/j.surge.2021.01.011

[20] Vidavsky N, Kunitake J, Estroff LA. Multiple pathways for pathological calcification in the human body. Adv Healthc Mater. 2021;10(4):e2001271. 33274854 10.1002/adhm.202001271 PMC8724004

[21] O’Grady S, Morgan MP. Microcalcifications in breast cancer: from pathophysiology to diagnosis and prognosis. Biochim Biophys Acta Rev Cancer. 2018;1869(2):310–20. 29684522 10.1016/j.bbcan.2018.04.006

[22] Hassler O. Microradiographic investigations of calcifications of the female breast. Cancer. 1969;23(5):1103–9. 4305104 10.1002/1097-0142(196905)23:5<1103::aid-cncr2820230514>3.0.co;2-7

[23] Frappart L, Boudeulle M, Boumendil J, Lin HC, Martinon I, Palayer C, et al. Structure and composition of microcalcifications in benign and malignant lesions of the breast: study by light microscopy, transmission and scanning electron microscopy, microprobe analysis, and X-ray diffraction. Hum Pathol. 1984;15(9):880–9. 6469237 10.1016/s0046-8177(84)80150-1

[24] Scimeca M, Giannini E, Antonacci C, Pistolese CA, Spagnoli LG, Bonanno E. Microcalcifications in breast cancer: an active phenomenon mediated by epithelial cells with mesenchymal characteristics. BMC Cancer. 2014;14:286. 24758513 10.1186/1471-2407-14-286 PMC4021315

[25] Tabar L, Tony Chen HH, Amy Yen MF, Tot T, Tung TH, Chen LS, et al. Mammographic tumor features can predict long-term outcomes reliably in women with 1-14-mm invasive breast carcinoma. Cancer. 2004;101(8):1745–59. 15386334 10.1002/cncr.20582

[26] Tsau HS, Yen AM, Fann JC, Wu WY, Yu CP, Chen SL, et al. Mammographic tumour appearance and triple-negative breast cancer associated with long-term prognosis of breast cancer death: a Swedish cohort study. Cancer Epidemiol. 2015;39(2):200–8. 25731718 10.1016/j.canep.2015.01.013

[27] Woodard GA, Ray KM, Joe BN, Price ER. Qualitative Radiogenomics: Association between Oncotype DX Test recurrence score and BI-RADS mammographic and breast MR Imaging features. Radiology. 2018;286(1):60–70. 28885890 10.1148/radiol.2017162333

[28] Nyante SJ, Lee SS, Benefield TS, Hoots TN, Henderson LM. The association between mammographic calcifications and breast cancer prognostic factors in a population-based registry cohort. Cancer. 2017;123(2):219–27. 27683209 10.1002/cncr.30281 PMC5287030

[29] Morgan MP, Cooke MM, Christopherson PA, Westfall PR, McCarthy GM. Calcium hydroxyapatite promotes mitogenesis and matrix metalloproteinase expression in human breast cancer cell lines. Mol Carcinog. 2001;32(3):111–7. 11746823 10.1002/mc.1070

[30] Bocca C, Ievolella M, Autelli R, Motta M, Mosso L, Torchio B, et al. Expression of Cox-2 in human breast cancer cells as a critical determinant of epithelial-to-mesenchymal transition and invasiveness. Expert Opin Ther Targets. 2014;18(2):121–35. 24325753 10.1517/14728222.2014.860447

